# A rapid adjunct to the postmortem diagnostic workup for right ventricular dysplasia

**DOI:** 10.11604/pamj.2023.44.164.31492

**Published:** 2023-04-06

**Authors:** Nasuhi Engin Aydin

**Affiliations:** 1 Department of Pathology, Izmir Katip Celebi University, Ataturk Hospital, Izmir, Turkey

**Keywords:** Cardiomyopathy, sudden death, autopsy, forensic

## Image in medicine

Right ventricular dysplasia (RVD) is characterized by fibrofatty replacement of the myocardium of the right ventricle and has been shown to cause sudden death. As also described in this case a very important feature that has been noted in all cases of RVD, is right ventricular (RV) wall thinning with fatty replacement. An observation that has been put forward for immediate postmortem opinion is that transillumination of the thinned myocardium could yield a suspicion for RVD as could be seen in this case of a young female college student who ingested a high dose of anti-depressive medication and who was first thought of suicidal attempt (A, B). The postmortem toxicological analysis was also negative for any lethal dose of medication. RVD or its synonym arrhythmogenic right ventricular cardiomyopathy (ARVC) was included in the 1995 classification of cardiomyopathies of the World Health Organization but was recently proposed to be included in a concept termed “arrhythmogenic cardiomyopathy” (AC).

**Figure 1:**
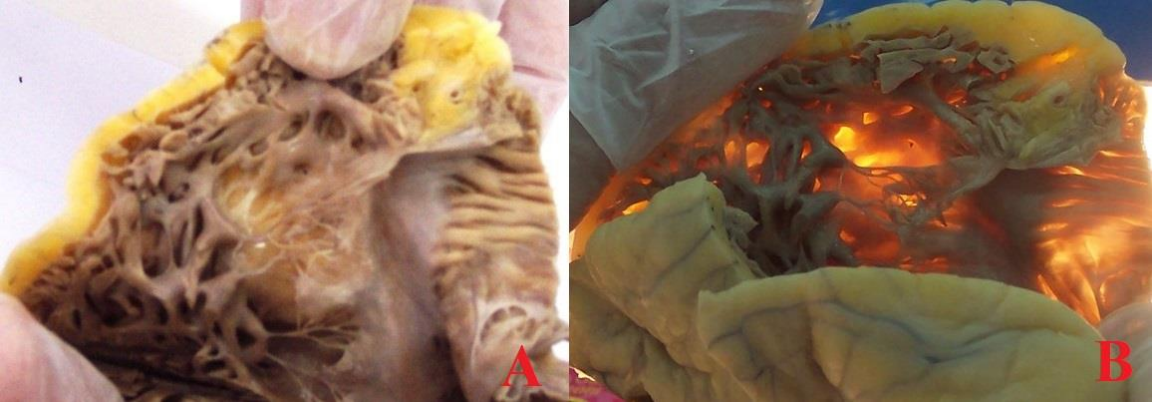
postmortem view of the right ventricle in a case of sudden death, with thinning and fatty replacement A) with remarkable positive transillumination B) due to the loss of the myocardial layer

